# The Roles of Exosomes in the Diagnose, Development and Therapeutic Resistance of Oral Squamous Cell Carcinoma

**DOI:** 10.3390/ijms24031968

**Published:** 2023-01-19

**Authors:** Shan Shi, Zi-Li Yu, Jun Jia

**Affiliations:** 1 The State Key Laboratory Breeding Base of Basic Science of Stomatology (Hubei-MOST) & Key Laboratory of Oral Biomedicine Ministry of Education, School and Hospital of Stomatology, Wuhan University, Wuhan 430079, China; 2 Department of Oral and Maxillofacial Surgery, School and Hospital of Stomatology, Wuhan University, Wuhan 430079, China

**Keywords:** exosome, oral squamous cell carcinoma, biomarker, therapeutic resistance

## Abstract

Oral cancer is one of the most common cancers worldwide, of which more than half of patients are diagnosed at a locally advanced stage with poor prognosis due to recurrence, metastasis and resistant to treatment. Thus, it is imperative to further explore the potential mechanism of development and drug resistance of oral cancer. Exosomes are small endosome-derived lipid nanoparticles that are released by cells. Since the cargoes of exosomes were inherited from their donor cells, the cargo profiles of exosomes can well recapitulate that of their donor cells. This is the theoretical basis of exosome-based liquid biopsy, providing a tool for early diagnosis of oral cancer. As an important intracellular bioactive cargo delivery vector, exosomes play a critical role in the development of oral cancer by transferring their cargoes to receipt cells. More importantly, recent studies have revealed that exosomes could induce therapy-resistance in oral cancer through multiple ways, including exosome-mediated drug efflux. In this review, we summarize and compare the role of exosomes in the diagnosis, development and therapy-resistant of oral cancer. We also highlight the clinical application of exosomes, and discuss the advantages and challenges of exosomes serving as predictive biomarker, therapy target and therapy vector in oral cancer.

## 1. Introduction

Oral cancer is one of the top 10 malignant tumors worldwide, leading to over 177,000 deaths annually [1]. More than 90% of oral cancer cases are histologically oral squamous cell carcinoma (OSCC). Accumulating evidence shows that early OSCC patients have a favorable prognosis, with 5-year survival rates up to 80% [2]. Unfortunately, more than half of OSCC patients are diagnosed at a locally advanced stage (Ⅲ/Ⅳ) [3]. Compared with their early counterparts, patients with OSCC in stage Ⅲ/Ⅳ had a significantly poor prognosis and 5-year survival rates less than 30% due to recurrence and metastasis [4]. In addition, despite progress in the available treatments for advanced OSCC [5,6,7,8], the 5-year survival rate has not improved significantly during the past decades. Therefore, it is a major concern to explore the potential mechanism of recurrence and metastasis of OSCC. In recent years, an increasing number of studies have demonstrated that exosomes were closely associated with the progression of various types of cancer including OSCC. 

Exosomes are small endosome-derived lipid nanoparticles that are released by both tumor cells and non-tumor cells. Exosomes carry different bioactive molecules and can deliver those cargoes to receipt cells to participate in many biological processes, including the recurrence and metastasis of cancer. Since the cargoes of exosomes are inherited from their donor cells, the cargo profiles of exosomes can well recapitulate that of their donor cells. This is the theoretical basis of exosome-based liquid biopsy. Previous studies [9], including ours [10,11,12], have shown the considerable difference of exosomes between OSCC patients and healthy donors, providing great potential for disease diagnosis. Exosomes are emerging as a new type of cancer biomarkers in recent decades. In addition, as an important intracellular bioactive cargoes delivery vector, exosomes play a critical role in the development of OSCC (e.g., recurrence, metastasis, immune response, angiogenesis and epithelial-mesenchymal transition) by transferring their cargoes to receipt cells [13]. Further, in recent decades, researches [14] have revealed that exosomal contents affect antitumor therapy by regulating sensitivity of the tumors to treatment, usually leading to resistance to therapy and subsequently recurrence and metastasis. In this review, we summarize the role of exosomes in the diagnosis, prognosis, development and treatment of OSCC. In addition, we also highlight the clinical application of exosomes, and discuss the advantages and challenges of exosomes in diagnosis, therapy target and therapy vector in OSCC.

## 2. Biogenesis and Classification of Exosomes

**Figure 1:**
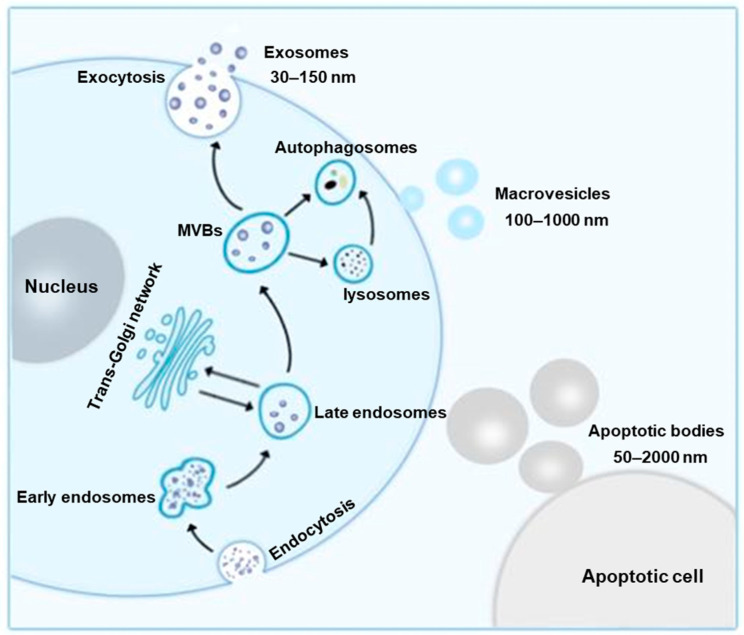
Biogenesis and classification of exosomes. Early endosomes are formed through the invagination of the plasma membrane. As endosomes mature, numerous multivesicular bodies (MVBs) can arise by the inward budding of the late endosomal limiting membrane. Finally, the MVBs can fuse with plasma membranes to release contained exosomes. Microvesicles are generated directly from the out budding of the plasma membrane. Apoptotic bodies are formed by apoptotic cell during shrinkage and death.

Extracellular vesicles (EVs) are nano-sized membranous vesicles comprising exosomes, microvesicles and apoptotic bodies. Exosomes, a small extracellular vesicle subtype (30–150 nm in diameter), are formed by activating endosomal pathway. As illustrated in Figure 1, early endosomes are formed through the invagination of the plasma membrane. As endosomes mature, numerous multivesicular bodies (MVBs) can arise by the inward budding of the late endosomal limiting membrane. Finally, the MVBs can fuse with plasma membranes to release contained exosomes or undergo intracellular degradation within lysosomes and autophagosomes. Microvesicles, the larger EVs fraction (100–1000 nm in diameter), are generated directly from the out budding or shedding of the plasma membrane. Apoptotic bodies, the largest EVs subtypes (50–2000 nm in diameter), are formed by apoptotic cell during shrinkage and death [15]. The biogenesis of EVs is represented schematically in Figure 1.

EVs are involved in the interaction between cells, whereas each subpopulation has different biofunctions. Exosomes, the critical mediators of intercellular communication, participate in various biological processes including cell adhesion [16,17], coagulation [18,19], immune response and cell transdifferentiation [20]. Exosomes are also involved in the development of multiple diseases [21,22], especially a variety of tumors [23]. Exosomes play an important role in regulating tumor initiation, growth and therapy. Similarly, microvesicles are involved in various pathophysiological processes. Apoptotic bodies are essential to maintain homeostasis in vivo [24,25]. In addition, different subpopulations of EVs exhibit high heterogeneity in their physical properties. The characteristics of different subsets of EVs are summarized in Table 1.

Regrettably, by virtue of the overlap size and difficult traceability, the taxonomic utility of EVs is limited. In addition, although increasing studies have sought to distinguish EVs subtypes [26,27], there is actually no single reliable marker for discriminating between exosomes and microvesicles. In 2018, the International Society for Extracellular Vesicles (ISEV) suggested that EVs could be subdivided into small extracellular vesicles (<200 nm) and large extracellular vesicles (>200 nm) [28]. Small extracellular vesicles (sEVs) are mainly composed of exosomes. Large extracellular vesicles (lEVs) are mixture of microvesicles and apoptotic bodies.

**Table 1:** Characteristics of different exosomes, microvesicles and apoptotic bodies.

Characteristic	Exosomes	Microvesicles	Apoptotic Bodies	References
Morphology	Cup-shape	Heterogenies	Heterogenies	[29]
Origin	Endosome	Plasma membrane	Plasma membrane	[30]
Size	30–150 nm	100–1000 nm	50–2000 nm	[30,31,32]
Content	1. Nucleic acid (small RNA, DNA) 2. Protein (functional protein) 3. Lipid (glycolipids, free fatty acids)	1. Nucleic acid (RNA, DNA) 2. Protein (functional protein, organelle protein) 3. Lipid (ceramides, sphingomyelins)	1. Nucleic acid (rRNA, DNA) 2. Protein (histone, organelle protein) 3. Lipid	[33,34]
Marker	Tetraspanin, TSG101, Alix	Unknown	Apoptosis-related protein	[35,36]
Function	Involved in various pathophysiological processes	Involved in various pathophysiological processes	Maintain the stability of the internal environment	[17,18,19,20,21,22,23,24]

## 3. Exosomes in the Diagnosis of OSCC

So far, tissue biopsy remains the gold standard for the diagnosis of oral cancer. Many OSCC are developed from different types of oral mucosal diseases including leukoplakia, erythema and lichen planus [37,38]. The process of carcinogenesis among different site of the same mucosal diseases is usually inconsistent. Thus, the diagnostic accuracy of biopsy, which is seriously affected by the sample sites of biopsy, is not absolute in these OSCC developed from oral mucosal diseases due to the limited size and different site of sample [39]. In addition, the development of OSCC from mucosal diseases is a prolonged process; it is difficult to decide when does the biopsy should be performed since it is unavoidable associated with invasion, pain and wounds. Thus, early diagnosis of OSCC that developed from mucosal diseases is highly challenging. There has been an increasing and sustained demand for new non-invasive, practical diagnostic strategies. Since exosomes inherit features from their mother cells in both physiological and pathological conditions, they show great potential to serve as diagnostic marker. Furthermore, they are widely present and remarkably stable in human biofluids including plasma and saliva. Exosome-based liquid biopsy has become a promising alternative for diagnosis and prognosis of OSCC (Table 2). 

### 3.1. Exosomes Derived from Saliva

Saliva is presented in the oral cavity and direct contacts with oral cancer, reflecting the relevant state of oral disease and providing the possibility for early diagnosis of OSCC. Ogawa et al. have demonstrated that exosomes are highly abundant in human saliva [56]. In our previous research, we found that the level of salivary microvesicles (SMVs) was significantly increased in OSCC patients. Further analysis found that the elevated SMVs was correlated with prognosis, staging and clinical outcomes [40]. The profile of salivary exosomal miRNA differs considerably between OSCC patients and healthy persons, which may be a promising option for OSCC diagnosis [57,58]. Using qRT-PCR analysis, researchers identified a marked increase of miR-24-3p [41], miR-512-3p [42], miR-412-3p [42] and miR-31 [43] in salivary exosomes from patients with OSCC. Notably, Gai et al. found that miR-302b-3p and miR-517b-3p were highly expressed in exosomes from OSCC patients [42], while undetectable in exosomes from healthy controls. In addition to miRNA, protein cargo in salivary exosomes could also serve as a potential biomarker in the diagnosis and prognosis of OSCC [44]. Fontana et al. found 365 differential protein expression in salivary exosomes isolated from healthy controls and OSCC patients with or without lymph node metastasis (LNM) by performing a proteome quantitative SWATH-MS analysis [45]. Other studies have demonstrated that exosomal protein was associated with immune response, providing new insights into OSCC prognosis [46]. Recently, Zlotogorski-Hurvitz et al. showed the especial IR spectrum of salivary exosomes contributed to the diagnosis of early stage OSCC [47].

### 3.2. Exosomes Derived from Plasma

Blood is the only fluid in direct contact with all organs and that transports important information throughout the whole bodies [59,60,61]. Many studies have demonstrated the concentration and contents of exosomes in blood vary significantly in pathophysiological conditions. Plasma-derived exosomes have become a promising alternative for the diagnosis of OSCC [57]. We previously reported the significantly increased level of circulating exosomes in OSCC patients compared with healthy donors [10]. Further analysis revealed that the elevated level of exosomes promotes a hypercoagulable state in OSCC via releasing inflammatory factors. Squamous cell carcinoma antigen (SCCA) was a potential marker for diagnosis of OSCC [62]. Yang et al. found SCCA in plasma-derived exosomes was remarkably elevated after saponin treatment [48]. In addition, Li et al., concluded that ApoA1, CXCL7, PF4V1 and F13A1 from serum exosomes could serve as novel diagnosis biomarkers for OSCC with lymph node metastasis [49]. Some scholars found that higher expression of miR-155 [50], miR-21 [50], miR-130a [51], CD63 [52] and CAV1 [52] in serum exosomes was associated with lower survival in oral cancer. They concluded that exosomal miR-155 and miR-21 contributed to tumorigenesis by inhibiting both PTEN and Bcl-6 expression [50]. On the contrary, other scholars proposed that exosomal protein (ALDH7A1, CAD, CANT1, GOT1, MTHFD1, PYGB and ASRS) [53], miRNA (miR-126 [50] and miR-146a [54]) and mtDNA [63] were negatively correlated with survival outcome. As described above, plasma-derived exosome is emerging as a novel diagnostic marker for OSCC.

### 3.3. Exosomes Derived from Other Origins

In addition to saliva and plasma, exosomes derived from drainage fluid also provide guiding significance for the diagnosis and prognosis of OSCC. Drainage fluid enters lymph circulation through lymphatic vessels, playing an important role in immunity [64]. Wang et al., found 313 differential protein expression in drainage fluid-derived exosomes isolated from OSCC patients with or without lymph node metastasis by performing a proteome quantitative analysis [55]. They suggested drainage fluid-derived exosomes may serve as a potential metastasis marker for OSCC.

## 4. Exosomes in the Development of OSCC

**Figure 2:**
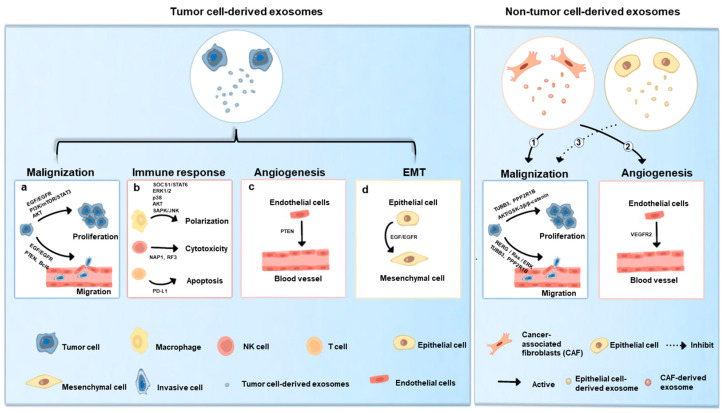
Exosomes in the development of OSCC. Tumor cell-derived exosomes (TEXs) support tumor development through promoting malignization of tumor cells (**a**), inhibiting antitumor immune responses (**b**), promoting angiogenesis (**c**) and promoting Epithelial-to-mesenchymal transition (EMT) (**d**). Cancer-associated fibroblasts (CAF)-derived exosomes contribute to tumor growth by promoting malignization of tumor cells (**1**) and angiogenesis (**2**). Epithelial cell-derived exosomes impair tumor growth through inhibiting malignization of tumor cells (**3**).

In addition to cancer diagnosis, researchers found that exosomes affected the development of oral cancer by transportation of their contents to target cells [41,65]. However, exosome in body fluids is a hybrid of exosome secreted from multiple cells including tumor cells and non-tumor cells (e.g., immune cells and fibroblasts). Exosomes are significantly diverse in their bioactive cargoes among cell types, leading to different effects on the development of cancer. Thus, it is vital to investigate the impacts of exosomes from different cell sources on OSCC. The functions of exosomes in tumor development are represented schematically in Figure 2.

### 4.1. Tumor Cell-Derived Exosomes in OSCC

#### 4.1.1. Exosomes-Mediated Malignization

Cancer cells could communicate with each other via tumor cell-derived exosomes (TEXs), affecting proliferation, migration and invasion of cells [66,67] and thereby prompting the malignization. Activation of EGF/EGFR signaling pathway promoted tumor progression and enhanced the malignant potential of OSCC cells through cellular uptake of TEXs [66]. Anti-EGFR agents may be effective for the treatment of patients with OSCC by blocking not only the direct EGF/EGFR signaling pathway but also uptake of TEXs through macropinocytosis. Studies have demonstrated that exosomal miRNA (miR-24-3p [41] and miR-21-5p [68]) remarkedly increased proliferation and viability of OSCC cells. The periodic circadian rhythm adjustment factor (PER1) is an important regulator in cell cycle [69]. Exosomal miR-24-3p could enhance cell proliferation by inhibiting PER1 expression [41]. Chen et al. concluded that exosomal miR-21-5p contributed to the growth rate of OSCC cells by regulating the PI3K/mTOR/STAT3 signaling pathway [68]. In addition, other researchers also found the increased cell invasiveness and migration in the recipient cells after co-incubation with TEXs. Exosomal miR-21 [50,70], miR-155 [50] and miR-200c-3p [71] played a supporting role in migration and invasion of tumor cells, which were significant processes for tumor metastasis. Li et al. found that tumor metastasis in mice was promoted by injection of miR-21-rich TEXs [70]. 

#### 4.1.2. Exosomes-Mediated Immune Response

TEXs can regulate immune response through direct interaction with immune cells, including Natural killer (NK) cells, T cells and macrophages. The NK cell, a major innate immune cell, exerts a crucial role in early innate immune response of tumor [72,73]. More recently, NK cells have been considered to demonstrate immune memory, playing an important role in adaptive immune responses to tumors [74]. Wang et al., revealed that TEXs increased the cytotoxicity of NK cells toward OSCC cells [75]. They concluded that NAP1, which was found enriched in TEXs, activated NK cells through increasing RF3 expression in the target cell. T cells are the major effector cells of antitumor immunity. Previous study has demonstrated that exosomal PD-L1 promoted tumor growth through inhibiting the activation and function of T cells [76]. Similarly, other researchers found exosomal PD-L1 could reduce T cell infiltration in OSCC [67]. Macrophages, important innate immune cells, can be roughly divided into antitumor M1-like phenotype and pro-tumor M2-like phenotype. Cai et al. found exosomal miR-29a-3p promoted tumor growth by inducing M2 polarization of macrophages in OSCC [77]. Tumor-associated macrophages (TAMs) are the major tumor-infiltration immune cells [78]. Increased TAM infiltration (especially M2-like phenotype) is correlated with frequent metastasis in OSCC patients [79,80]. Increasing evidence have suggested that TEXs promoted tumor growth and metastasis by inducing M2 polarization of TAMs in OSCC. Exosomal CDC37 [81], HSP90 [81] and CMTM6 [82] play an important role in M2 polarization of TAMs. Moreover, exosomal THBS1 induces M1 polarization of TAMs, promoting future malignant phenotypes of OSCC [83]. 

#### 4.1.3. Exosomes-Mediated Angiogenesis

Angiogenesis is the generation of new blood vessels, which is dependent on the proliferation, migration and invasion of vascular endothelial cells (ECs). Angiogenesis is a crucial process for tumor growth and metastasis [84]. Exosomes released by OSCC cells were able to be internalized by vascular endothelial cells, thereby enhancing angiogenesis. Our previous study also found that EVs in the circulation [11] and tumor tissues [85] of OSCC patients could facilitate angiogenesis by enhancing the proliferation, migration and invasion of ECs. Yan et al. further demonstrated that miR-130b-3p in OSCC cell-derived exosomes promoted the formation of new blood vessels via inhibiting PTEN expression [86]. 

#### 4.1.4. Exosomes-Mediated Epithelial-to-Mesenchymal Transition

Epithelial-to-Mesenchymal Transition (EMT) is a key cellular process during which epithelial cells gain the mesenchymal phenotype. This process has important biological significance in embryonic development [87]. However, EMT is abnormally activated in diverse tumors, leading to malignant metastasis [88]. Additionally, the role of EMT in drug resistance [89] and immune escape [90] has been emphasized during recent decades. Unfortunately, the mechanism underlying EMT activation in tumors remains unclear. Nevertheless, a growing number of researches found TEXs-induced EMT activation in various tumors, including OSCC [91,92,93]. Fujiwara et al. reported the abnormal activation of EMT in OSCC after co-incubation with EGFR-rich TEXs [94]. Moreover, the activation of EMT was damaged by co-incubating with both EGFR-rich TEXs and EGFR inhibitor, suggesting that exosomal EGFR was a crucial regulator of EMT activation.

### 4.2. Non-Tumor Cell-Derived Exosomes in OSCC

In addition to TEXs, exosomes secreted by non-tumor cells (e.g., epithelial cells, fibroblasts) were able to regulate tumor development. Cui et al. uncovered exosomal miR-200c from epithelial cells inhibited the proliferation, migration and invasion of tumor cells [95]. The inhibitory effect of exosomal miR-200c could be reversed by the miR-200c inhibitor. However, the result was contradictory to exosomal miR-200c-3p originating from tumor cells, because a previous study [71] found the increased invasiveness in recipient tumor cells. Cancer-associated fibroblasts (CAFs) are one of the main components in tumor stroma [96]. Numerous studies have demonstrated that CAFs are associated with poor prognosis of tumors [97,98]. In OSCC, CAF-derived exosomes could promote tumor growth and metastasis by increasing migration [99], invasion [99] proliferation [100] and angiogenesis of cells [101]. Among them, exosomal miR-382-5p [99], miR-34a-5p [100] and heparan sulfate proteoglycans [101] play a crucial role in this process.

## 5. Exosomes in the Treatment of OSCC

**Figure 3:**
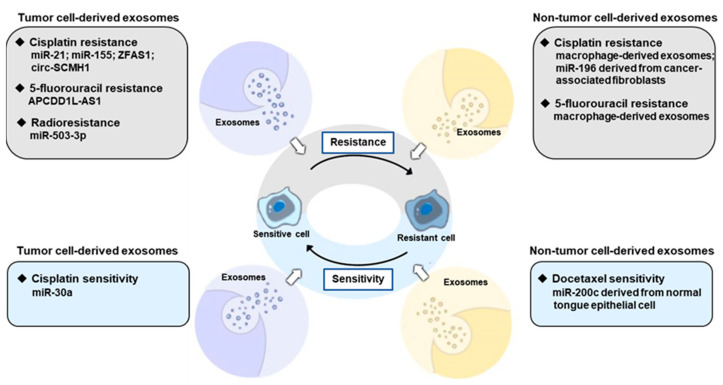
Exosomes in the therapeutic resistance of OSCC. Exosomes derived from both tumor cells and non-tumor cells can regulate therapy resistance of OSCC cells through multiple ways.

Currently, surgery, chemotherapy, radiotherapy and immunotherapy are recognized as the main therapeutic regimens for OSCC patients. Despite various attempts, the overall survival rates of OSCC patients have not substantially improved in the last decades [102]. The treatment resistance is a major reason for treatment failure in OSCC. In addition to decreasing response rate, the acquisition of treatment resistance often results in tumor relapse, leading to poor prognosis in OSCC patients. Thus, further exploration of the potential mechanism of drug resistance is urgently needed. Researchers found that exosomes could induce therapy-resistant in proper context [103,104]. In Figure 3, we schematize mechanisms involved in exosome-mediated therapy-resistant.

### 5.1. Tumor Cell-Derived Exosomes-Mediated Therapy Resistant

TEXs could induce resistant to drug therapy and radiotherapy. Cisplatin (CDDP) and 5-fluorouracil (5-FU) were first-line chemotherapeutic drugs in OSCC treatment. Drug efflux constitutes an important mechanism in drug resistance of tumor cells. Recent studies demonstrated that tumor cells could increase drug efflux and thus decrease the accumulation of anticancer drugs by the secretion of exosomes. A proton pump inhibitor enhances the accumulation of drugs and the susceptibility of tumor cells to CDDP by reducing TEXs secretion [105]. Other authors have reported the supporting function of exosomal miR-21 [68,106], miR-155 [107], ZFAS1 [108] and circ-SCMH1 [109] in CDDP resistance. Kulkarni et al. found decreased expression of circulating exosomal miR-30a in OSCC patients with recurrence, and revealed the inhibitory effect of TEXs-derived miR-30a in CDDP resistance [110]. In addition to CDDP resistance, researchers [66,111] also found increased tolerance of 5-FU in OSCC cells after co-incubation with TEXs. Li et al. proposed exosomal APCDD1L-AS1 conferred 5-FU resistant to 5-FU sensitive cells via miR-1224-5p/nuclear receptor binding SET domain protein 2 (NSD2) axis [111]. In addition, few studies [112] also demonstrated the enhanced radioresistance in radiosensitive OSCC cells after co-incubating with miR-503-3p-rich exosomes. 

### 5.2. Non-Tumor Cell-Derived Exosomes-Mediated Therapy Resistant

Similarly, exosomes derived from non-tumor cells also affected therapy efficacy. miR-200c in exosomes derived from normal tongue epithelial cells impaired drug resistant (Docetaxel) of OSCC cells [95]. On the contrary, Tomita et al. found macrophage-derived exosomes conferred drug resistance (including Cisplatin and 5-FU) to OSCC cells through the activation AKT/GSK-3β signaling pathway in vivo [113]. Qin et al. also found exosomal miR-196 released from CAF remarkably enhances CDDP resistance in OSCC cells (CAL27 cell) [114]. 

## 6. Applications and Challenges of Exosomes in OSCC

### 6.1. Application as Predictive Biomarker

Exosomes inherit features from their mother cells, thus providing a possibility for tumor surveillance. Exosomes are widely present in human biofluids and tissues. Among them, exosomes derived from plasma and saliva have been reported to be reliable markers for OSCC. In contrast to traumatic biopsy, the collection of saliva and blood is procedurally easy and non-invasive, thereby reducing infection rate and other side effects. In addition, collection of exosomes is not obviously impacted by cancer site. This process avoids interference caused by improper sampling, and meanwhile brings new possibilities for detection of deep tumors. In addition, the time point of sample collection can be selected throughout the treatment period, and is not limited to preoperative. Hence, exosome-mediated tumor prediction provides a practical method for real-time monitoring of tumor activity during antitumor therapy. Further, exosome-based detection is not in direct contact with tumor masse, avoiding irritation of tumor tissue, thus reducing the risk of metastasis of cancer caused by invasive biopsy. Moreover, compared to the liquid-based biopsy (e.g., free nucleic acid), the lipid membrane of exosomes protects exosomal contents from degradation, providing a more reliable outcome.

Exosomes are emerging as new type of cancer biomarker; however, some obstacles toward the practical application of exosomes in the clinic exist. Cargo within exosomes differs considerably among healthy donors and OSCC patients with and without good prognosis. However, the differential exosomal contents exhibit high heterogeneity. There is no consensus on the selection of differential content and definition of detectable difference. In addition, non-tumor cell derived exosomes are abundant in biofluids [115,116,117], exacerbating the complexity of molecular source, thereby bringing obstacles for the identification of harbored markers and low abundance markers [118]. In contrast to plasma, saliva is considered as a special fluid associated with oral cancer. To further standardize the application of exosomes, it is vital to summarize the characters and challenges of salivary exosomes.

Saliva mainly concentrates in the oral cavity and is in direct contact with oral cancer, reflecting the relevant state of oral disease, providing the possibility for early oral cancer diagnosis. In contrast to plasma, salivary exosome-secreting cells are mainly tumor cells and oral mucosal cells, increasing concentrations of exosome-based biomarkers, thus enhancing the sensitivity and specificity for tumor detection. Additionally, salivary exosomal proteins are relatively simple [119], further facilitating the identification of exosome-based biomarkers. With regard to multimarker detection, our previous research [12] designed a one-step strategy for diagnosing oral ulcers and oral cancer. By using magnetic capture, EpCAM^+^ (oral cancer derived) and CD45^+^ exosomes (oral ulcers derived) are easily separated.

To further assess clinical applications of saliva-derived exosomes, multiple problems need to be considered. First, saliva-derived exosomes directly interact with the extraoral environment and are affected by various environmental factors. The human oral cavity contains complex microbial communities. Keller et al. [120] demonstrated that bacteria interfere with the expression of exosomal protein and secretion of exosomes. The intraoral environment (such as PH, temperature) could also be changed by daily activities such as drinking, eating and speaking. Second, the isolation method of salivary exosomes needs further optimization. At present, ultracentrifugation is the most commonly used method. However, saliva remains sticky after ultracentrifugation, which may impact functional properties of exosomes. Hence, proper pretreatment is needed for the saliva supernatant. Notably, Ohshiro et al. [121] proposed that initial saliva processing affected detection of tumor marker. The amount of saliva can also be affected by antitumor therapy, bringing difficulties for collection of exosomes. For example, irreversible hyposalivation is common in oral cancer patients after radiotherapy.

### 6.2. Application as Therapy Target

**Figure 4:**
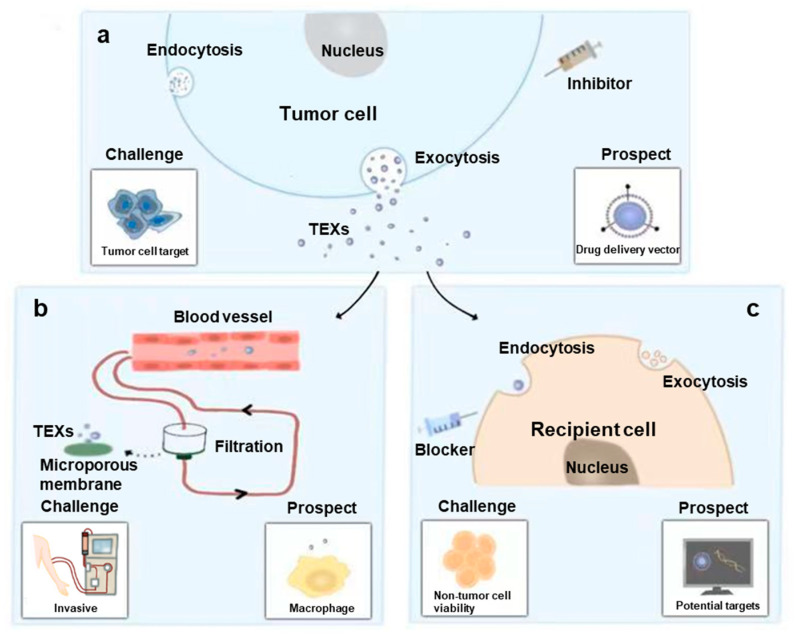
Challenges and prospects for TEX-targeted therapies. TEX-targeted therapies can be divided in three ways: inhibit biogenesis, tracking and secretion of TEXs (**a**), facilitate TEXs clearance (**b**) and block function of TEXs (**c**). For TEX inhibiting therapy, tumor-targeted drug delivery vectors will be a promising option to promote the tumor-targeted ability of exosomal inhibitor. For TEXs clearance therapy, side effects are inevitable due to invasive procedure. The novel strategy should be developed to further improve macrophage-mediated TEXs clearance. For TEXs blocking therapy, potential targets should be explored to decrease the impact on non-tumor cells.

In this review, we summarize the role of exosomes in tumor development and treatment. TEXs enhanced tumor growth by promoting proliferation, migration, invasion and angiogenesis of tumor cells, while inhibiting antitumor immunity. Moreover, TEXs contributed to tumor progression by inducing therapeutic resistant. Hence, TEX-targeted therapies could be considered as promising options in OSCC treatment. Generally speaking, three pathways are included: inhibit biogenesis, tracking and secretion of TEXs, facilitate TEXs clearance and block function of TEXs. These strategies are represented schematically in Figure 4.

#### 6.2.1. Inhibit Biogenesis, Tracking and Secretion of TEXs

The accurate mechanism of biogenesis, tracking and secretion of TEXs still remains unclear. Despite this, three crucial biological processes for exosome biogenesis have been identified: formation of endosome, degradation of MVB and release of MVB. Currently, various inhibitors targeting these processes have been reported. In previous studies, formation of ILV mainly depends on the ESCRT complexes and accessory proteins [122,123]; an ESCRT-independent [124,125] pattern also exists. Datta et al. [126] found the decreased formation of ESCRT-dependent exosomes after stimulation with manumycin A. Ceramide provided an important foundation for ESCRT-independent exosomes biogenesis [127]. GW4869, a noncompetitive inhibitor of nSMAse, effectively inhibited exosomes production by blocking ceramide synthesis. Additionally, the role of sulfisoxazole in lysosomal targeting of MVB inhibited exosomes generation [128]. Furthermore, Catalano et al. [129] proposed the inhibitory activity of cytochalasin D on release of MVB. 

However, the majority of the drugs do not specifically target tumor cells, thus impairing the secretion of non-tumor cell-derived exosomes. Hence, a novel strategy to confer tumor-targeted ability is desperately required. By virtue of specific targeting property, nanosized drug delivery systems are considered as emerging options for cancer treatment. Studies have revealed that the accumulation of drugs in tumor cells can be enhanced by tumor-targeted nanoparticles [130]. Inhibition of TEXs biogenesis will be achieved specially when combining exosome inhibitors with the tumor-targeted drug delivery systems. In addition, since its target is enriched in tumor cell [131], manumycin A appeared to exert a stronger inhibitory effect on TEXs biogenesis [126]. Therefore, to develop better tumor-targeted agents, more studies are required to explore the underlying mechanisms of exosome generation, especially the unique mechanism in tumor cells. 

#### 6.2.2. Facilitate Clearance of TEXs

Previous research [132] summarized the important application of hemofiltration in TEXs clearance. TEXs are selectively captured and transported to blood circulation, and subsequently removed through the filtration of the microporous membrane. Mesoporous silica nanoparticles (MSNs) exhibit excellent biosafety. Xie et al. [133] functionalized MSNs with EGFR-targeting aptamers (MSN-AP) for specific binding to EGFR-rich exosomes. The bound exosomes are delivered and finally eliminated in the small intestine. However, hemofiltration, a traumatic treatment, inevitably exacerbates infection risk of patients. In addition, the ubiquitously clearance of both TEXs and non-tumor cell-derived exosomes hinder the future application of hemofiltration. With regard to MSN-AP, their applications also have limitations due to the nonhomogeneous expression of EGFR on tumor cells. 

Previous studies reported that exosomes were principally metabolized in the liver, spleen and kidney, engulfed by mononuclear phagocyte system [134]. TEXs can enhance their circulation time by inhibiting macrophage-mediated phagocytosis [135]. Shimizu et al. [136] reported that the overexpressed CD47 (“don’t eat me” signal) on TEXs protect them from phagocytosis, extending their lifetime in circulation. In addition, PD-L1, which was recently shown to be another “don’t eat me” signal, was also highly expressed on exosomes released by various cancer cells. Therefore, blocking the “don’t eat me” signal on TEXs will be a promising strategy to enhance macrophage-mediated TEXs clearance.

#### 6.2.3. Block Function of TEXs by Inhibiting Endocytosis

**Figure 5:**
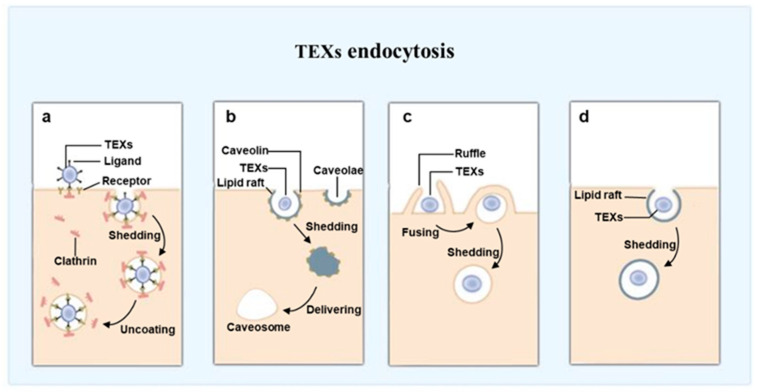
Forms of TEXs endocytosis. TEXs endocytosis can roughly be divided into four forms: clathrin-mediated endocytosis (**a**), caveolin-dependent endocytosis (**b**), micropinocytosis (**c**) and lipid raft-mediated endocytosis (**d**).

Endocytosis of exosome by receive cells is the key process for exosome to perform biological functions [137,138]. There are multiple pathways that participate in exosome internalization, but the detailed mechanism remains unclear. Exosomes endocytosis can roughly be divided into four forms: clathrin-mediated endocytosis, micropinocytosis, caveolin-dependent endocytosis and lipid raft-mediated endocytosis [139,140,141] (Figure 5). With regard to TEXs internalization, Christianson et al. [142] found TEXs uptake dependent largely on HSPGs (heparan sulfate proteoglycans). Christianson et al. [143] summarized the role of HSPGs in endocytosis. They demonstrated that HSPG endocytosis is not regulated by a specific pathway. Heparan sulfate analogues can attenuate tumor growth by regulating TEXs uptake [144]. In addition, Sasabe et al. [66] proposed that EGF may trigger uptake of TEXs by OSCC cells in a time and dose-dependent manner. They found EGFR inhibitors suppress tumor progression through micropinocytosis-mediated TEXs internalization.

Consequently, blocking endocytosis of TEXs is an excellent prospect for antitumor therapy in the future. However, TEXs internalization is not completely suppressed by blockers. More potential targets of TEXs internalization need future investigation. Additionally, blocker may also affect cell viability and survival. The influence on non-tumor cells should be emphasized at working concentrations. In addition, TEXs can also regulate tumor growth through uptake-independent mechanisms [145]. Thus, blocking function of TEXs by inhibiting endocytosis is still in its infancy.

### 6.3. Application as Therapy Vector

By virtue of the excellent biocompatibility, specific targeting property and favorable stability in human biofluids, exosomes were considered as promising drug delivery carriers. In our previous study [146], we constructed functional plasma-derived exosomes via efficient antitumor siRNA loading with electroporation. Compared with the control group, functional exosomes treated group had a better potency in OSCC treatment. Moreover, we monitored the distribution and metabolism, and revealed the natural tumor targeting of exosomes isolated from the peripheral blood of parents with OSCC. Other researchers [147,148] also indicated that TEXs could be used as vectors for drug delivery. Kase et al. [149] demonstrated that the selection of exosomes for tumor-targeting was a high priority. They developed a nanoplatform by embedding therapeutic siRNA into exosome isolated from normal fibroblasts. In addition, HEK293T cell-derived exosomes have also been reported as drug delivery vehicle in OSCC treatment [150]. In addition, many studies [151,152] have reported the controlled release of exosomes. Controlled degradation of hydrogels plays an essential role in this phenomenon.

In recent decades, exosomes have made notable progress in therapy vector [153,154,155]. However, there are still some issues which hamper their universal applications in clinical practice. Two major challenges facing their use as therapy vector are high production and efficient drug loading of exosomes. Additionally, therapeutic exosomes are mainly used in animal experiments at present. Dosages of exosomes exhibit high heterogeneity in their animal model, administration method and cellular origin [156]. For future clinical translation, complete guidelines on therapeutic exosome should be developed. In addition, efficiency of exosome-mediated targeted therapy is associated with origin of exosome [157]. TEXs are considered as promising candidates for therapeutic vectors [158]. However, the caution that TEXs contribute to tumor growth via multiple pathways should be taken when using TEXs as therapeutic vectors.

## 7. Conclusions

Exosomes have wide applicability in oral cancer diagnosis and treatment. Although tissue biopsy remains the gold standard for diagnosis of oral cancer, exosomes derived from saliva or plasma are considered as promising diagnostic biomarkers due to their homogeneity, convenience and safety. However, there is no consensus on the selection of a standard biomarker or a definition of detectable difference. In addition, targeting TEXs therapy is an emerging option in OSCC treatment; however, this novel strategy is still in its infancy. It is an inevitable issue to impair non-tumor cell-derived exosomes. Furthermore, exosomes have made notable progress as a therapy vector; however, commercial acquisition of exosomes is still a major challenge. Despite the excellent prospects, there is still a long way to realize clinical application of exosomes.

## Figures and Tables

**Table 2:** Potential applications of exosomes from different samples collected from OSCC patients and healthy donors using different detecting methods.

Sources	Methods	Findings	Clinical Application	References
Saliva	FCM	Increased number in OSCC; higher ratio of apoptotic to non-apoptotic exosomes in lower survival	Diagnosis Prognosis	[40]
Saliva	qRT-PCR	Higher expression of miR-24-3p in OSCC	Diagnosis	[41]
Saliva	qRT-PCR	miR-302b-3p and miR-517b-3p only expressed in OSCC; miR-512-3p and miR-412-3p expression level increased in OSCC	Diagnosis	[42]
Saliva	qRT-PCR	Higher expression of miR-31 in OSCC	Diagnosis	[43]
Saliva	Proteome analysis	Proteins expression level were correlated with OSCC diagnosis and prognosis	Diagnosis Prognosis	[44,45,46]
Saliva	IR spectrum	Differential IR spectrum in OSCC patients compared with normal donors	Diagnosis	[47]
Plasma	FCM	Increased number in OSCC	Diagnosis	[10]
Plasma	Chemiluminescence immunoassay analyzer	Higher expression of SCCA in OSCC	Diagnosis	[48]
Plasma	Proteome analysis	Expression levels of 4 proteins were correlated with metastasis OSCC	Diagnosis	[49]
Plasma	qRT-PCR	Higher expression of miR-155 and miR-21 in OSCC; lower expression of miR-126 in OSCC with lower survival	Diagnosis Prognosis	[50]
Plasma	qRT-PCR	Higher expression of miR-130a in OSCC; higher expression of miR-130a in OSCC with lower survival	Diagnosis Prognosis	[51]
Plasma	ELISA	Decreased level of CD63^+^ exosomes or CAV-1^+^ exosomes in OSCC with higher survival	Prognosis	[52]
Plasma	Proteome analysis	7 proteins expression level decreased in OSCC with lower survival	Prognosis	[53]
Plasma	miRNA-Seq	Lower expression of miR-146a was correlated with OSCC malignancy	Prognosis	[54]
Drainage fluid	Proteome analysis	365 proteins expression level are correlated to lymph node metastasis in OSCC	Prognosis	[55]

## Data Availability

Not applicable.
